# RabGAP AS160/TBC1D4 deficiency increases long-chain fatty acid transport but has little additional effect on obesity and metabolic syndrome in ADMSCs-derived adipocytes of morbidly obese women

**DOI:** 10.3389/fmolb.2023.1232159

**Published:** 2023-08-03

**Authors:** Agnieszka Mikłosz, Bartłomiej Łukaszuk, Elżbieta Supruniuk, Kamil Grubczak, Magdalena Kusaczuk, Adrian Chabowski

**Affiliations:** ^1^ Department of Physiology, Medical University of Bialystok, Bialystok, Poland; ^2^ Department of Regenerative Medicine and Immune Regulation, Medical University of Bialystok, Bialystok, Poland; ^3^ Department of Pharmaceutical Biochemistry, Medical University of Bialystok, Bialystok, Poland

**Keywords:** adipose tissue, ADMSCs, AS160/TBC1D4, diacylglycerols, free fatty acids, fatty acid transport proteins, obesity, triacylglycerols

## Abstract

The Akt substrate of 160 kDa (AS160), also known as TBC1 domain family member 4 (TBC1D4), represents a crucial regulator of insulin-stimulated glucose uptake in skeletal muscle and adipose tissue. Recent evidence suggests that AS160/TBC1D4 may also control the cellular entry of long-chain fatty acids (LCFAs), resulting in changes to the lipid profile of muscles and fat cells in lean subjects. However, there are virtually no data on AS160/TBC1D4 expression and its modulatory role in lipid metabolism in the adipocytes from morbidly obese individuals of different metabolic status. In this study, we evaluated the effect of the three main factors, i.e., AS160 silencing, obesity, and metabolic syndrome on lipid uptake and profile in fully differentiated adipocytes derived from mesenchymal stem cells (ADMSCs) of lean and obese (with/without metabolic syndrome) postmenopausal women. Additionally, we tested possible interactions between the explanatory variables. In general, obesity translated into a greater content of fatty acid transporters (especially CD36/SR-B2 and SLC27A4/FATP4) and boosted accumulation of all the examined lipid fractions, i.e., triacylglycerols (TAGs), diacylglycerols (DAGs), and free fatty acids (FFAs). The aforementioned were further enhanced by metabolic syndrome. Moreover, AS160 deficiency also increased the abundance of SLC27A4/FATP4 and CD36/SR-B2, especially on the cell surface of the adipocytes derived from ADMSCs of subcutaneous deposit. This was further accompanied by increased LCFA (palmitic acid) uptake. Despite the aforementioned, AS160 silencing seemed unable to significantly affect the phenotype of the adipocytes stemming from obese patients with respect to their cellular lipid profile as we observed virtually no changes in TAG, DAG, and FFA contents when compared to cells with the reference level of proteins. Nevertheless, knockdown of AS160 stimulated fatty acid oxidation, which may indicate that adaptive mechanisms counteract excessive lipid accumulation. At the same time, adipocytes of visceral origin were rather insensitive to the applied intervention.

## 1 Introduction

The Akt substrate of 160 kDa (AS160), also known as TBC1 domain family member 4 (TBC1D4), is an Rab-GTPase–activating protein (RabGAP) that governs insulin-stimulated redistribution of glucose transporter 4 (GLUT4) from intracellular localization to the plasma membrane (Mafakheri et al., 2018). Thus, AS160 acts as a negative regulator of the aforementioned GLUT4 translocation process. In line with that notion, a previous investigation has demonstrated that reduced amounts of AS160 translate into smaller inhibitory influence of the protein on Rab and therefore greater plasmalemma expression of the glucose transporter (Sano et al., 2007). Interestingly, another member of the TBC1 domain family, i.e., TBC1D1, performs analogous actions with respect to GLUT4 trafficking (Sakamoto and Holman, 2008). Moreover, the recent decade has brought in evidence on the role of both proteins in the control of fatty acids' intracellular influx. Samovski et al. (2012), for instance, described the influence of AS160 silencing on the increased redistribution of CD36/SR-B2 to the sarcolemma of HL-1 cardiomyocytes. Other groups have confirmed that the deficiency of either TBC1D1 or TBC1D4 causes an enhanced expression of fatty acid handling proteins and a boosted influx of fatty acids into a skeletal muscle cell (Mikłosz et al., 2016; Benninghoff et al., 2020). Recently, we have demonstrated that the knockdown of AS160, but not TBC1D1, leads to an increased plasmalemmal and total CD36/SR-B2 content in the adipocytes differentiated from adipose-derived mesenchymal stem cells (ADMSCs) of lean women (Mikłosz et al., 2021). However, scientific understanding of the topic in human adipocytes is still far from being complete.

Fatty acids (FAs) are an important component of many biological systems. The aforementioned impose tight control on their transmembrane entry and subsequent cellular handling. The bulk of fatty acid uptake is mediated by a variety of different fatty acid transporters and binding proteins (Hao et al., 2020). The most important of which are cluster of differentiation 36 (CD36) [also known as scavenger receptor class B protein (SR-B2)] and two members of the SLC27 family of FA transport proteins (FATPs), i.e., SLC27A1/FATP1 and SLC27A4/FATP4. Among the aforementioned biomolecules, CD36/SR-B2 is a major fatty acid transporter present in metabolically active tissues (Pepino et al., 2014). Coburn et al. (2000) demonstrated that the adipocytes of CD36/SR-B2 knockout mice showed a pronounced reduction (−60% to −70%) in cellular fatty acid uptake (Coburn et al., 2000). Importantly, humans with CD36/SR-B2 deficiency (Pro90Ser CD36 mutation) also exhibited a markedly decreased FA uptake in the adipose tissue when compared to control subjects (Hames et al., 2014). On the other hand, chronic lipid overload in the blood (hyperlipidemia) is believed to shift CD36/SR-B2 toward the cell surface in the ectopic tissues (skeletal muscles, heart, and liver). The relocation predisposes the cells to excessive lipid accumulation, thus triggering pathophysiological impairments observed in obesity and type 2 diabetes mellitus (Aguer et al., 2011).

Obesity is a metabolic disease centered around white adipose tissue (WAT) dysfunction. Importantly, the epidemiological data suggest that its prevalence has increased dramatically within the last few decades reaching pandemic proportions (Mikłosz et al., 2022b). WAT is a prominent energy reservoir, distributed in two main depots—subcutaneous (SAT) and visceral adipose tissue (VAT). Both the depots differ not only with respect to their location but also with regard to their metabolic characteristics, e.g., various adipokine secretory profiles or distinct responsiveness to neuroendocrine stimuli (Reyes-Farias et al., 2021). A notable example of the discussed differences is the fact that in humans the accretion of body fat by SAT enlargement is considered to play a protective role against obesity’s adverse health complication. Whereas, the expansion of VAT is recognized as a risk factor for metabolic disorders (Hwang and Kim, 2019). Although mature adipocytes constitute ∼90% of the adipose tissue volume, they are not the only component of physiological importance. The remaining 10% comprise the so-called stromal vascular fraction (SVF) that contains, among others, stem cells, preadipocytes, fibroblasts, or epithelial cells (Lee et al., 2013). Paradoxically, a number of reports have indicated that the underrepresented part of the tissue may be a major determinant of the differences observed between the discussed fat depots (Peinado et al., 2010). Adipose-derived mesenchymal stem cells (ADMSCs) can undergo differentiation into adipocytes, thus they constitute an almost unlimited source of mature adipose cells that serve as a base for tissue growth in obese individuals (Silva et al., 2015). Most likely, WAT depot–specific metabolic characteristics originate from the distinct adipose progenitor cell properties (Baglioni et al., 2012; Mikłosz et al., 2022b). Previous research have demonstrated that ADMSCs derived from subcutaneous locations (subADMSCs) display a much higher proliferation rate and adipogenic potential than those derived from the visceral areas (visADMSCs) (Baglioni et al., 2012; Badimon and Cubedo, 2017). This could explain the observed *in vivo* propensity of visceral fat to amass lipids by their accumulation inside the existing mature cells (hypertrophy) rather than by generating new ones (hyperplasia) (Mikłosz et al., 2022a).

In recent years, scientific investigation on ADMSCs has gained some momentum. So far, the researchers have explored their therapeutic potential toward improvement of insulin resistance, dyslipidemia, and restoration of liver functions (Cao et al., 2015; Liao et al., 2016; Ishida et al., 2020; Jaber et al., 2021; Mikłosz and Chabowski, 2023). In comparison to the ADMSCs themselves, studies on adipocytes derived from stem cells are virtually nonexistent. This is quite surprising since the progenitor cells offer a good source of mature adipocytes that enable scientists to study the properties of the fat cells per se, i.e., in isolation from the potentially obscuring influences of the body’s internal environment. Lately, we demonstrated that the adipocytes differentiated from the mesenchymal stem cells of obese patients display a range of phenotypic characteristics that distinguish them from their lean counterparts (Mikłosz et al., 2022a). For example, we noticed that morbid obesity of donor subjects translated into a greater CD36/SR-B2 protein content (total and/or cell surface) in mature adipocytes derived from subADMSCs. This in turn resulted in a greater palmitic acid uptake and lipids (TAG and DAG) accumulation in the cells. Surprisingly, the aforementioned changes were accompanied by an increased amount of AS160 in the adipocytes (Mikłosz et al., 2022a). In fact, similar increments have been observed *in vivo* in the adipose tissue of obese type 2 diabetic patients by Stafeev et al. (2019). Moreover, a previous study by Wu et al., (2014) demonstrated that AS160 contributes to lipid droplets' fusion and growth (Wu et al., 2014). In line with that notion, such a buildup in the AS160 protein content could explain the changes observed by us, i.e., the accumulation of lipids in voluminous vacuoles in the adipocytes differentiated from ADMSCs of obese individuals (Mikłosz et al., 2022a).

In the present study, we address the question of whether AS160 protein deficiency affects fatty acid trafficking and lipid metabolism in the *in vitro*–derived adipocytes with respect to the metabolic state of the donor and location of the adipose tissue. Accordingly, we obtained ADMSCs from subcutaneous and visceral WAT of normal weight and morbidly obese subjects and differentiated them into mature adipocytes. Using a human ADMSCs-derived adipocyte culture model, we assessed the expression and cellular localization of fatty acid–handling proteins and lipid profile. Additionally, we tested possible interactions between the explanatory variables, i.e., 1) AS160 silencing with obesity and 2) AS160 silencing with metabolic syndrome.

## 2 Materials and methods

### 2.1 Patients

The mesenchymal stem cells of the white adipose tissue were acquired from the subcutaneous (abdominal region) and visceral (omental region) depots from postmenopausal female subjects who underwent laparoscopic cholecystectomy or elective bariatric surgery at the first Department of General and Endocrine Surgery at the University Clinical Hospital in Białystok. The age-matched female individuals were classified into normal weight (BMI 19–24.9 kg/m^2^) or morbidly obese (BMI ≥40 kg/m^2^) subjects. The patients with obesity were further divided into two subgroups, i.e., individuals without metabolic syndrome (*n* = 4) and individuals with metabolic syndrome (*n* = 4). The diagnosis of the metabolic syndrome was performed based on the National Institutes of Health's criteria (MayoClinic, 2023). Briefly, the female patients had to fulfill at least three of the following traits: 1) waist circumference >102 [cm]; 2) blood triacylglycerol level of 150 [mg/dL] or higher; 3) blood HDL cholesterol level <50 [mg/dL]; 4) blood pressure of 130/85 [mmHg] or higher; and 5) fasting blood glucose level of 100 [mg/dL] or higher. The enrolled participants underwent clinical examination, anthropometric measurements, and appropriate laboratory tests (Table 1). The lean and morbidly obese patients with a positive medical history of acute inflammatory diseases and malignancy were excluded from the study. The study was approved by the Ethics Committee of the Medical University of Bialystok (permission R-I-002/187/2017) and was carried out in accordance with the Declaration of Helsinki and the Guidelines for Good Clinical Practice. All patients gave their informed consent for inclusion and participation in the study.

**TABLE 1:** Clinical characteristics of the lean and morbidly obese women (adipose tissue donors).

	Lean	Obese (−)	Obese (+)
**Age [yrs]**	61.0 ± 3.367	61.25 ± 2.63	62.0 ± 3.162
**BMI [kg/m** ^**2**^ **]**	23.33 ± 2.043	42.43 ± 2.243*	50.12 ± 4.511*^
**WHR**	0.84 ± 0.143	0.92 ± 0.029	0.92 ± 0.025
**Glucose [mg/dL**]	79.75 ± 10.243	96.75 ± 12.894	105.0 ± 4.83*
**Insulin**	6.99 ± 0.672	19.88 ± 2.622*	27.5 ± 7.148*
**HOMA-IR**	1.36 ± 0.076	4.74 ± 0.787*	7.17 ± 2.108*
**CRP [mg/L]**	5.78 ± 0.435	9.49 ± 0.848*	12.58 ± 2.457*
**Systolic pressure [mmHg]**	113.75 ± 4.787	118.75 ± 11.087	153.75 ± 9.465*
**Diastolic pressure [mmHg]**	77.5 ± 5.0	86.25 ± 4.787	92.5 ± 5.0*
**ALT [IU/L]**	26.5 ± 4.041	38.75 ± 6.344*	31.25 ± 1.893
**AST [IU/L]**	21.0 ± 2.0	18.5 ± 3.697	32.25 ± 8.221^
**Cholesterol [mg/dL]**	162.75 ± 6.702	163.25 ± 11.529	199.25 ± 57.996
**HDL [mg/dL]**	59.25 ± 6.238	49.25 ± 4.272*	37.0 ± 12.41*
**LDL [mg/dL]**	107.75 ± 1.5	116.5 ± 9.147	133.25 ± 35.929
**TAG [mg/dL]**	142.5 ± 4.655	149.5 ± 6.455	175.0 ± 19.201*
**RBC [mln/mm** ^**3**^ **]**	4.77 ± 0.149	4.62 ± 0.405	4.91 ± 0.285
**Hb [g/dL]**	13.62 ± 1.081	13.77 ± 0.892	12.88 ± 0.793
**WBC [thous./mm** ^**3**^ **]**	8.08 ± 1.962	7.44 ± 0.668	7.63 ± 1.666
**Platelets [thous./mm** ^**3**^ **]**	294.75 ± 23.128	275.25 ± 50.129	273.5 ± 11.902

Data are presented as mean and standard deviation. *p* < 0.05; * indicates significant differences *vs* lean patients; ^ indicates significant differences *vs* obese (−) group.

ALT, alanine transaminase; AST, aspartate transaminase; BMI, body mass index; CRP, C-reactive protein; DP, diastolic pressure; HDL, high-density lipoprotein; HGB, hemoglobin; HOMA-IR, homeostatic model assessment of insulin resistance; INR, international normalized ratio; LDL, low-density lipoprotein; PLT, platelet count; RBC, red blood cell count; SP, systolic pressure; TAG, triacylglycerol; WBC, white blood cell count; and WHR, waist–hip ratio.

### 2.2 Mesenchymal stem cell isolation and expansion

Immediately following the dissection, the samples were placed in phosphate-buffered saline (PBS, PAN-Biotech, Aidenbach, Germany) and transported to the laboratory for further processing. The ADMSCs were isolated according to the protocol described previously (Mikłosz et al., 2021). Briefly, the adipose tissue was extensively washed in PBS, minced with scissors and scalpel, and treated with collagenase (250 U/mL collagenase NB 4G Proved Grade, Serva, Heidelberg, Germany) for 1 h at 37°C. The digestion process was stopped by adding Dulbecco’s Modified Eagle Medium (DMEM, PAN-Biotech, Aidenbach, Germany) equaling five volumes of the initial digestion volume. The resulting suspension was filtered through a 500 µm strainer in order to remove mature adipocytes from the stromal vascular fraction (SVF) and centrifuged for 10 min at 600*g*. Next, the pellet was resuspended in erythrocyte lysis buffer (Thermo Fisher Scientific, Waltham, MA, United States), filtered through a 200 µm strainer and then a 20 µm strainer, and centrifuged again for 5 min at 600*g*. Following the second centrifugation, the ADMSCs-rich pellet was suspended in a mesenchymal stem cells medium containing growth supplements (MSCM, ScienCell Research Laboratories, Carlsbad, CA, United States), 5% fetal bovine serum (FBS, Thermo Fisher Scientific, Waltham, MA, United States), and antibiotics (PAN-Biotech, Aidenbach, Germany). The cultured medium was replaced every 2–3 days, and after they reached 80%–90% confluence, the cells were detached using TrypLE (Thermo Fisher Scientific, Waltham, MA, United States), counted and cryopreserved in freezing medium (Stem-Cell banker DMSO Free, Takara Bio, Mountain View, CA, United States), and stored in liquid nitrogen for further procedures.

In order to check whether the isolated cells satisfied the minimal criteria for defining multipotent mesenchymal stem cells, we checked the ADMSCs' phenotype and their multi-lineage differentiation potential. The phenotype of the isolated ADMSCs was confirmed using flow cytometry, and they were stained using fluorochrome-conjugated monoclonal antibodies: anti-CD73, anti-CD90, anti-CD105, anti-CD45, anti-CD133, anti-Lineage Cocktail 1 (CD3, CD14, CD16, CD19, CD20, CD56) (BD Bioscience). Phenotyping of the ADMSCs was performed in accordance with previously implemented protocol (Mikłosz et al., 2021).

### 2.3 Evaluation of cell phenotype—flow cytometric analysis

The acquired ADMSCs were subjected to staining with monoclonal antibodies targeting anti-FATP1 (ACSVL5; mouse anti-human) (R&D Systems), anti-FATP4 (ACSVL4; rabbit anti-human), and anti-CD36/SR-B2 (CD36/SR-B2; rabbit anti-human) (Abcam). Immunostaining was preceded by stratification of the cells into non-permeabilized and permeabilized groups (FACS Permeabilizing Solution 2, BD Bioscience), allowing the detection of extracellular only or together with intracellular proteins, respectively. Incubation of ADMSCs with antibodies (at room temperature, in dark) was followed by washing steps in phosphate-buffered saline without calcium and magnesium (PBS w/o Ca/Mg; Corning). Subsequently, secondary detection antibodies were used that included goat anti-mouse (Alexa Fluor 647) (Invitrogen) and goat anti-rabbit (Alexa Fluor 488). After incubation and additional washing steps, the cells were fixed using CellFIX (BD Biosciences) and stored at 4°C. Data were acquired using the FACSCalibur Flow Cytometer (BD Biosciences; San Jose, CA, United States). The FlowJo software (Tree Star Inc.; Ashland, OR, United States) was implemented for flow cytometric data processing. ADMSCs were initially distinguished morphologically on the basis of forward scatter (FSC; relative size) and side scatter (SSC; relative granularity/complexity). The gating strategy of the selected proteins was based on unstained and FMO controls. The results were presented as selected marker frequencies, extracellularly or extra- and intracellularly within the ADMSCs' population.

### 2.4 Adipogenic differentiation of human ADMSCs

All experiments were performed only in the first 2–4 cell passages. The adipogenic differentiation was induced by culturing the human ADMSCs with the Mesenchymal Stem Cell Adipogenic Differentiation Medium (MADM, ScienCell Research Laboratories, Carlsbad, CA, United States). The progress of adipogenesis was monitored by the microscopic observation of lipid vacuoles in the cells. The cells were maintained in the differentiation medium until all cells acquired fully mature adipocyte morphology (from 14 up to 21 days).

Mature ADMSCs-derived adipocytes were stained with Oil Red O (ORO, Sigma-Aldrich, St. Louis, MO, United States) dye to identify lipid vacuoles that were subsequently quantified by spectrometric analysis. Briefly, cell monolayers were rinsed with PBS and fixed with 10% formalin (Sigma-Aldrich, St. Louis, MO, United States) for 30 min at RT. After rinsing with PBS, adipocytes were stained with ORO solution for 1 h, washed, and subsequently imaged using an inverted microscope (Olympus, magnification ×400). Afterward, the absorbance of the extracted dye was measured at 510 nm using a microplate reader (Synergy H1 Hybrid Reader, BioTek, Santa Clara, CA, United States). The undifferentiated ADMSCs stained with Oil Red O served as the control.

### 2.5 RNA interference (RNAi)–mediated silencing in human adipocytes differentiated from ADMSCs

Fully differentiated ADMSCs-derived adipocytes were transfected using the Viromer GREEN transfection reagent (Lipocalyx, Halle, Germany) according to the manufacturer’s instruction. Briefly, siRNA targeting AS160/TBC1D4 at a concentration of 25 nM (Silencer Select Pre-designed siRNAs for AS160/TBC1D4—ID: s19140 and ID: s19142) and non-targeting control siRNA (Silencer Select Negative Control #1 siRNA) were used. The standard complexation protocol was employed using Viromer GREEN (Lipocalyx) according to the manufacturer's instruction.

### 2.6 Immunoblotting

The ADMSCs-derived adipocytes were lysed in RIPA buffer containing protease and phosphatase inhibitors (Roche Diagnostics GmbH, Mannheim, Germany). The protein content in the lysates was measured using the BCA method with bovine serum albumin (fatty acid–free, Sigma-Aldrich, St. Louis, MO, United States) as the standard. Before protein separation using SDS-PAGE (the same amounts of protein, i.e., 30 μg was loaded on Criterion TGX Stain-Free Precast Gels, Bio-Rad, Hercules, CA, United States) and being transferred to the PVDF membrane, the samples were denatured in Laemmli buffer (Bio-Rad, Hercules, CA, United States). Finally, the membranes with the transferred proteins were blocked in 5% non-fat dry milk in Tris-buffered saline–Tween and incubated with the corresponding primary antibodies: AS160 (Merck Millipore, CA, United States), DGAT1, β-HAD, ATGL, GAPDH (Santa Cruz Biotechnology, Inc., Dallas, TX, United States), and FASN (Abcam, Cambridge, UK). Binding of the primary antibody was detected after incubation by using HRP-conjugated secondary antibody and then Clarity or Clarity Max Western ECL substrates (Bio-Rad, Hercules, CA, United States). Protein bands were quantified densitometrically by using the ChemiDoc Imaging System (Bio-Rad, Hercules, CA, United States), and their expression was normalized to GAPDH reference protein expression.

### 2.7 RNA isolation and real-time polymerase chain reaction

Total RNA was isolated using the TRIzol Reagent (Sigma-Aldrich, St. Louis, MO, United States) with DNase I treatment according to the manufacturer's instructions. Spectrophotometric measurements (A260/A280) were carried out to evaluate the quantity and quality of the extracted RNA. Afterward, the RNA was reverse-transcribed into cDNA using the EvoScript Universal cDNA Master kit (Roche Molecular Systems, Boston, MA, United States). Amplification of the product was performed using FastStart Essential DNA Green Master (Roche Molecular Systems) as the detection dye using the LightCycler 96 System Real-Time thermal cycler (Roche, Mannheim, Germany). The following reaction parameters were applied: initial denaturation at 94°C for 10 min, followed by 45 cycles of denaturation at 94°C for 15 s, annealing at 57°C–62°C for 15 s, and extension at 72°C for 15 s. Primer sequences for AS160, CD36/SR-B2, FATP1, FATP4, and housekeeping RPL13A have been described in our recent studies (Mikłosz et al., 2022a). Gene expression was analyzed using the relative quantification method modified by Pfaffl (2001).

### 2.8 9,10-[^3^H]-Palmitic acid uptake

Palmitic acid uptake was evaluated according to the procedure by Chavez and Summers (2003). ADMSCs-derived adipocytes were starved in a serum-free medium for 3 h prior to experiment initiation. To start the uptake assay, a Krebs-Ringer HEPES buffer containing palmitic acid (Sigma-Aldrich, St. Louis, MO, United States) bound to bovine serum albumin (fatty acid–free, Sigma-Aldrich, St. Louis, MO, United States) and radiolabeled 9,10-^3^H palmitic acid (1 μCi/mL, Perkin Elmer, Shelton, CT, United States) was added to each well for 5 min at 37°C/5% CO_2_. Then, the plates were placed on ice, and each well was intensively washed with ice-cold PBS buffer. The residual washing buffer was completely aspirated, and the cells were lysed in protein lysis buffer. Half of the lysates were used for liquid scintillation counting, and the rest were used for protein measurement. Radioactivity was measured using a Packard TRI-CARB 1900 TR scintillation counter and normalized to protein concentrations.

### 2.9 Measurement of intracellular lipid content (gas liquid chromatography)

Adipocytes' lipid content (FFA, DAG, and TAG) was analyzed using the gas liquid chromatography (GLC) method. Briefly, lipids were extracted in chloroform–methanol solution (2:1 vol/vol) and then separated by using thin-layer chromatography (TLC) on silica gel plates (Silica Plate 60, 0.25 mm; Merck, Darmstadt, Germany). Individual fatty acids (FAs) from each fraction were methylated, and fatty acid methyl esters (FAMEs) were identified and quantified according to the standard retention times. The samples were analyzed byy using the Hawlett-Packard 5890 Series II gas chromatograph and HP-INNOWax capillary column. The lipid concentration was expressed in nanomoles per milligram of protein.

### 2.10 Statistics

The obtained data were preprocessed in MS Excel. The subsequent statistical analysis of the results was performed in GraphPad Prism (software version: 8.2.1). Three-way ANOVA was applied to examine the effects of the main factors (AS160 silencing, obesity, and metabolic syndrome) on the investigated parameters. Additionally, our model tested for possible interactions between the discussed explanatory variables, i.e., 1) AS160 silencing with obesity and 2) AS160 silencing with metabolic syndrome. The analysis was followed by the *post hoc* Tukey HSD test. As a cutoff level for statistical significance, the value of 0.05 was chosen (*p* < 0.05). The results of the analyses were put into tables and placed alongside the appropriate bar plots, and the data presented as mean ± SD.

To account for possible unspecific effects of the silencing process (e.g., the vector used for siRNA delivery), we employed a placebo group (cells transfected with a non-targeting siRNA). The results of the gene knockdown were compared to this group.

## 3 Results

### 3.1 Immunophenotypic characterization of expanded human ADMSCs

**FIGURE 1:**
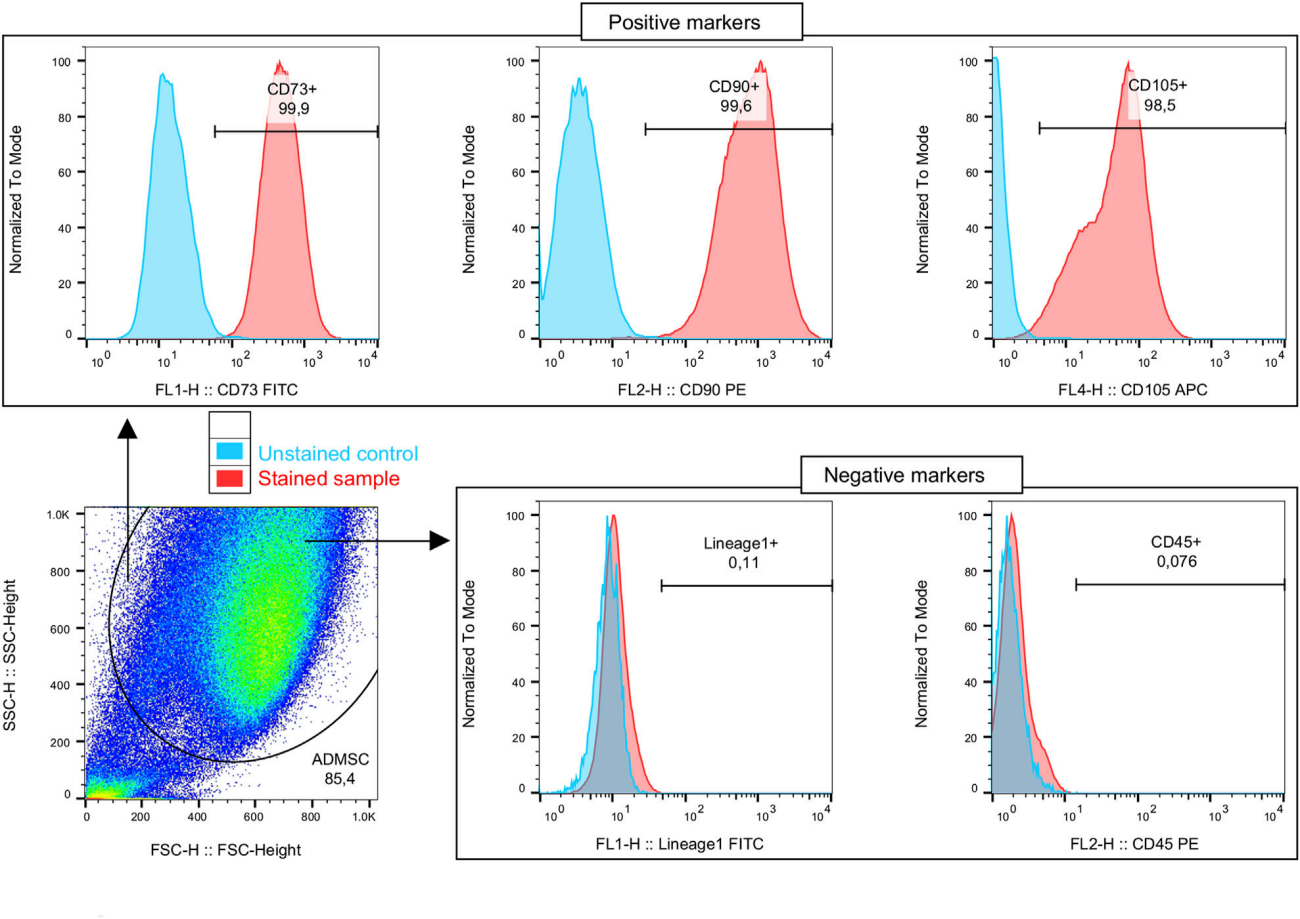
Immunophenotyping of the isolated adipose-derived mesenchymal stem cells (ADMSCs). Cells are initially distinguished on the basis of morphological properties (forward and side scatter—relative size and granularity/complexity, respectively). The ADMSCs' phenotype is confirmed on the basis of characteristic positive markers set presence: CD73, CD90, and CD105; and exclusion of other immune cells, with negative markers: CD45, Lineage1 (CD3, CD14, CD16, CD19, CD20, and CD56).

The flow cytometry analysis showed that ADMSCs derived from both subcutaneous and visceral adipose tissues display relatively comparable percentages of cells positive for cell surface markers, which are characteristic of mesenchymal stem cells. Namely, the analyzed cells expressed high levels of several antigens such as CD73, CD90, and CD105 at approximately 99% of the cells but lacked CD45 and lineage markers, which were used to identify potential contaminants, such as hematopoietic stem cells (Figure 1). Moreover, as formerly reported, we confirmed the multi-lineage differentiation potential of the isolated ADMSCs into osteocytes, chondrocytes, and adipocytes (Mikłosz et al., 2021). The aforementioned together with the ability to adhere to plastic after isolation from tissues and in *in vitro* culture indicate that the cells met all the requirements proposed by the International Society for Cellular Therapy (ISCT) (Dominici et al., 2006). Moreover, proper differentiation into adipocytes was determined using light microscopy and Oil Red O staining.

### 3.2 Transfection efficiency

**FIGURE 2:**
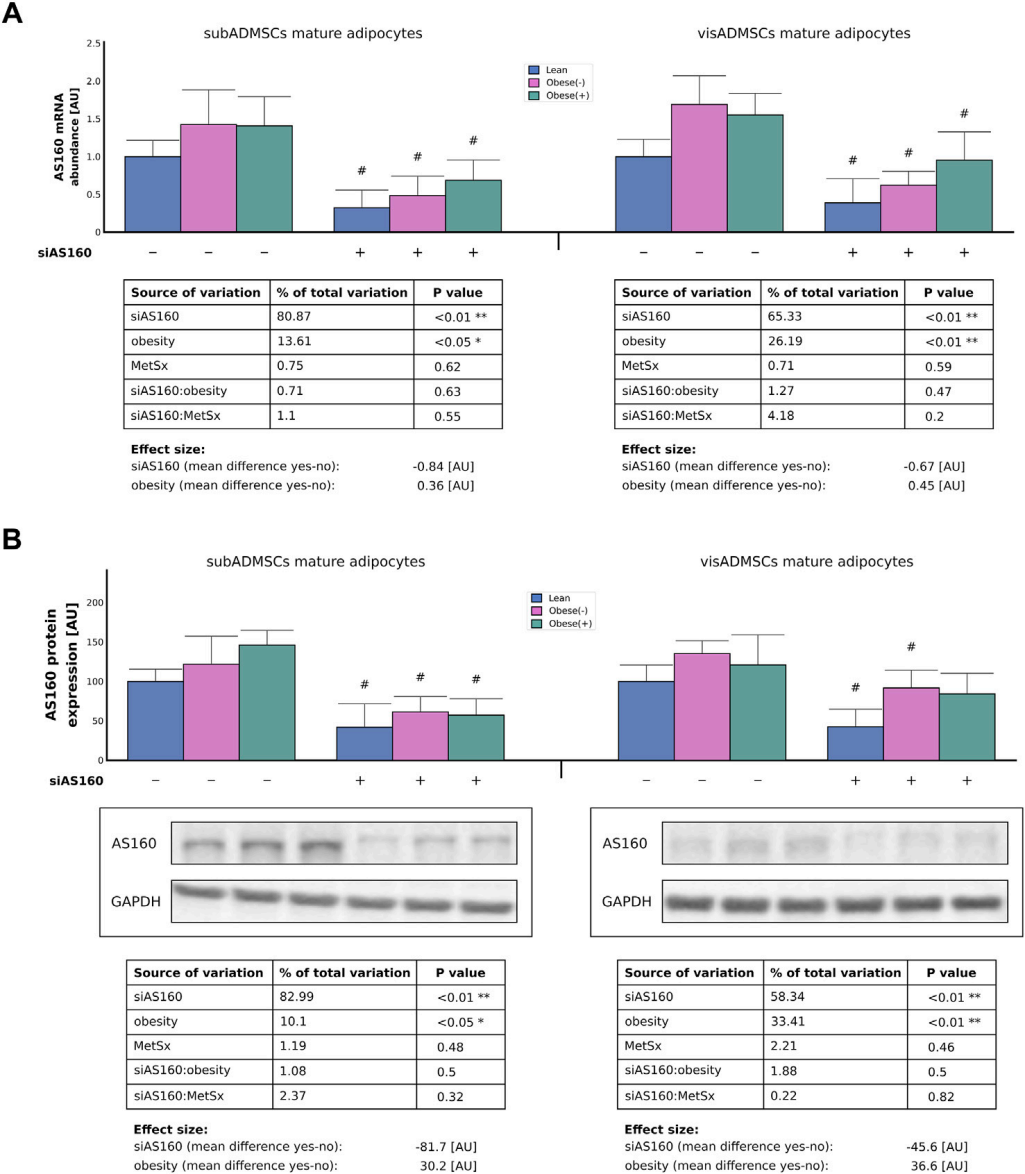
Real-time PCR **(A)** and Western blot **(B)** quantification of AS160 knockdown efficiency in adipocytes differentiated from adipose-derived mesenchymal stem cells of lean and morbidly obese women. Data are presented as mean ± SD. Values are expressed in arbitrary units, i.e., lean non-targeting siRNA group was set at 1 [AU] (mRNA) or 100 [AU] (protein). # difference between the cells transfected with siRNA targeting AS160 and scrambled siRNA (negative control) within the metabolic status of the donor patient; *p* < 0.05; *n* = 4 for each study group (measurements taken in duplicate). Representative Western blot images are shown. Glyceraldehyde-3-phosphate dehydrogenase served as protein loading control. Designations of the groups: NC, negative control containing non-targeting siRNA fragment; siAS160, knockdown of AS160; obese (−), obese without metabolic syndrome; and obese (+), obese with metabolic syndrome.

To investigate the importance of AS160 in fatty acid transport and metabolism, subADMSCs- and visADMSCs-derived adipocytes of lean and morbidly obese women were transfected with siRNA against AS160. For easier interpretation of the obtained results, the expression levels in the reference groups were assumed to be 1 [AU] (gene level) or 100 [AU] (protein content). The real-time PCR analysis confirmed that both AS160 gene silencing (∼81% and ∼65% of data variability, *p* < 0.01, for the subADMSCs and visADMSCs mature adipocytes, respectively) and obesity (∼14% and 26% of data variability, *p* < 0.05 and *p* < 0.01, for subADMSCs and visADMSCs, respectively) had an impact on the transcriptional level of AS160 in the mature adipocytes derived from ADMSCs (Figure 2A). On average, silencing of AS160 reduced the expression of its gene by 0.84 [AU] in the adipocytes derived from subADMSCs and by 0.67 [AU] in the case of the cells obtained from visADMSCs. Of note, obesity translated into a greater level of the gene transcript.

Similarly, both gene silencing (83% and 58% of data variability, *p* < 0.01, for subADMSCs and visADMSCs, respectively) and obesity (10% and 33% of data variability, *p* < 0.05 and *p* < 0.01, for subADMSCs and visADMSCs, respectively) had an impact on the total AS160 protein expression (Figure 2B). On average, AS160 silencing reduced the expression of its protein by 82 [AU] in the adipocytes that originated from subADMSCs, and by 46 [AU] in the case of the adipocytes of visADMSC ancestry. On the other hand, obesity contributed to a greater level of the protein (Figure 2B). In line with the aforementioned, AS160 was successfully knocked down using the small interfering RNA regardless of the donor patients' metabolic status (lean or obese without or with metabolic syndrome) or the tissue of origin (subcutaneous or visceral depot).

### 3.3 Loss of AS160 akt substrate enhances rate of cellular fatty acid (FA) uptake in adipocytes derived from ADMSCs of lean and morbidly obese individuals

To determine whether AS160 deficiency affected fatty acid transport, we measured the surface and total protein abundance of fatty acid handling proteins. Generally, the rate of cellular fatty acid (FA) uptake is short- and long-term regulated. Short-term regulation occurs through the reversible recycling of FA transporters from intracellular compartments to the plasma membrane, while long-term regulation of the FA uptake, which occurs in obesity (high fatty acid supply), requires changes in the gene transcription and/or protein abundance.

#### 3.3.1 Gene transcript and protein abundance of fatty acid transport proteins

**FIGURE 3:**
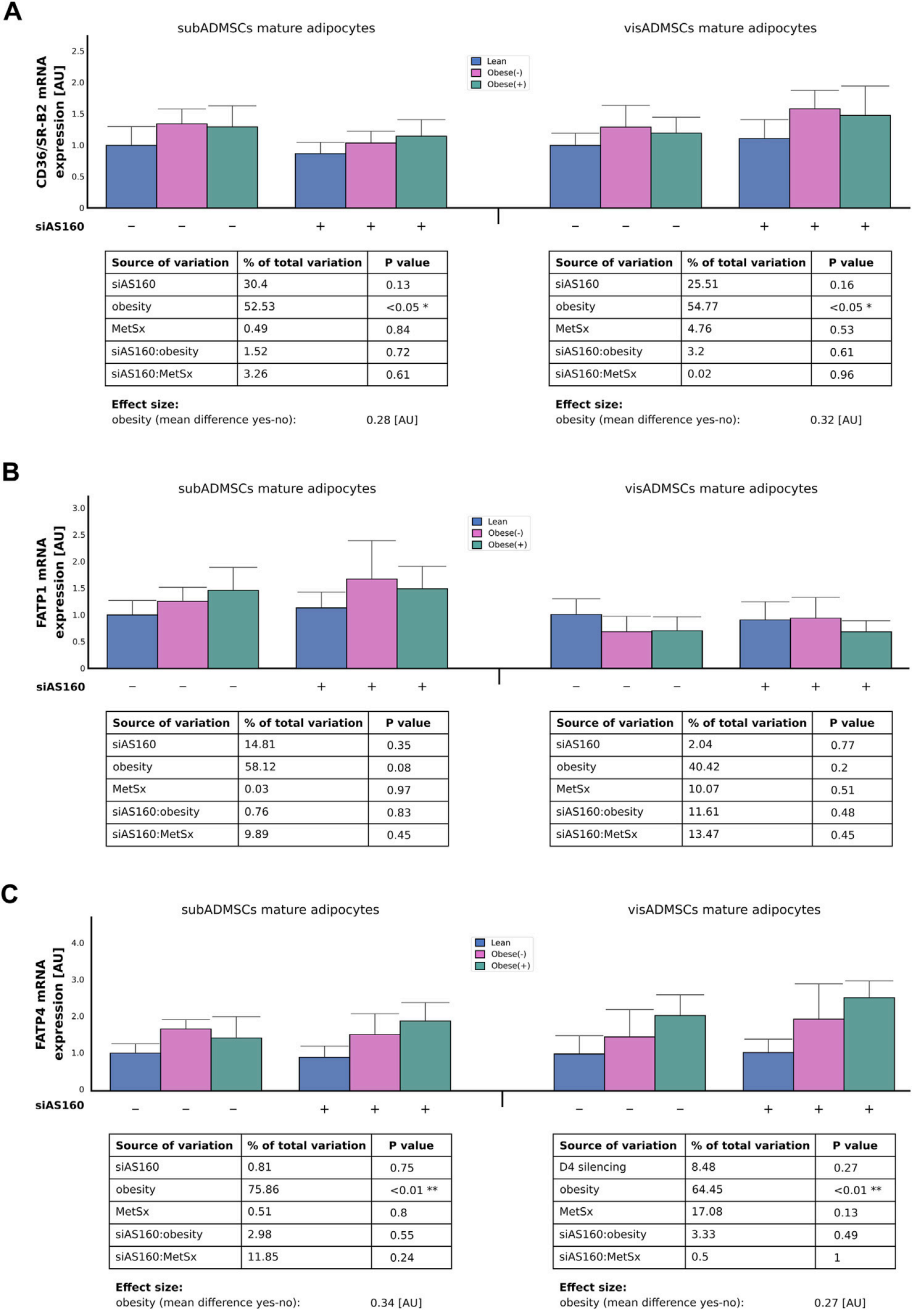
Transcript level of fatty acid transporters, i.e., CD36/SR-B2 **(A)**, FATP1 **(B)**, and FATP4 **(C)** in AS160 knocked down mature adipocytes differentiated from ADMSCs of patients with different metabolic status. Data are presented as mean ± SD. Values are expressed in arbitrary units, i.e., lean non-targeting siRNA group was set at 1 [AU]. # Difference between the cells transfected with siRNA targeting AS160 and scrambled siRNA (negative control) within the metabolic status of the donor patient; *p* < 0.05; *n* = 4 for each study group (measurements taken in duplicate). Designations of the groups: NC, negative control containing non-targeting siRNA fragment; siAS160, knockdown of AS160; obese (−), obese without metabolic syndrome; and obese (+), obese with metabolic syndrome.

**FIGURE 4:**
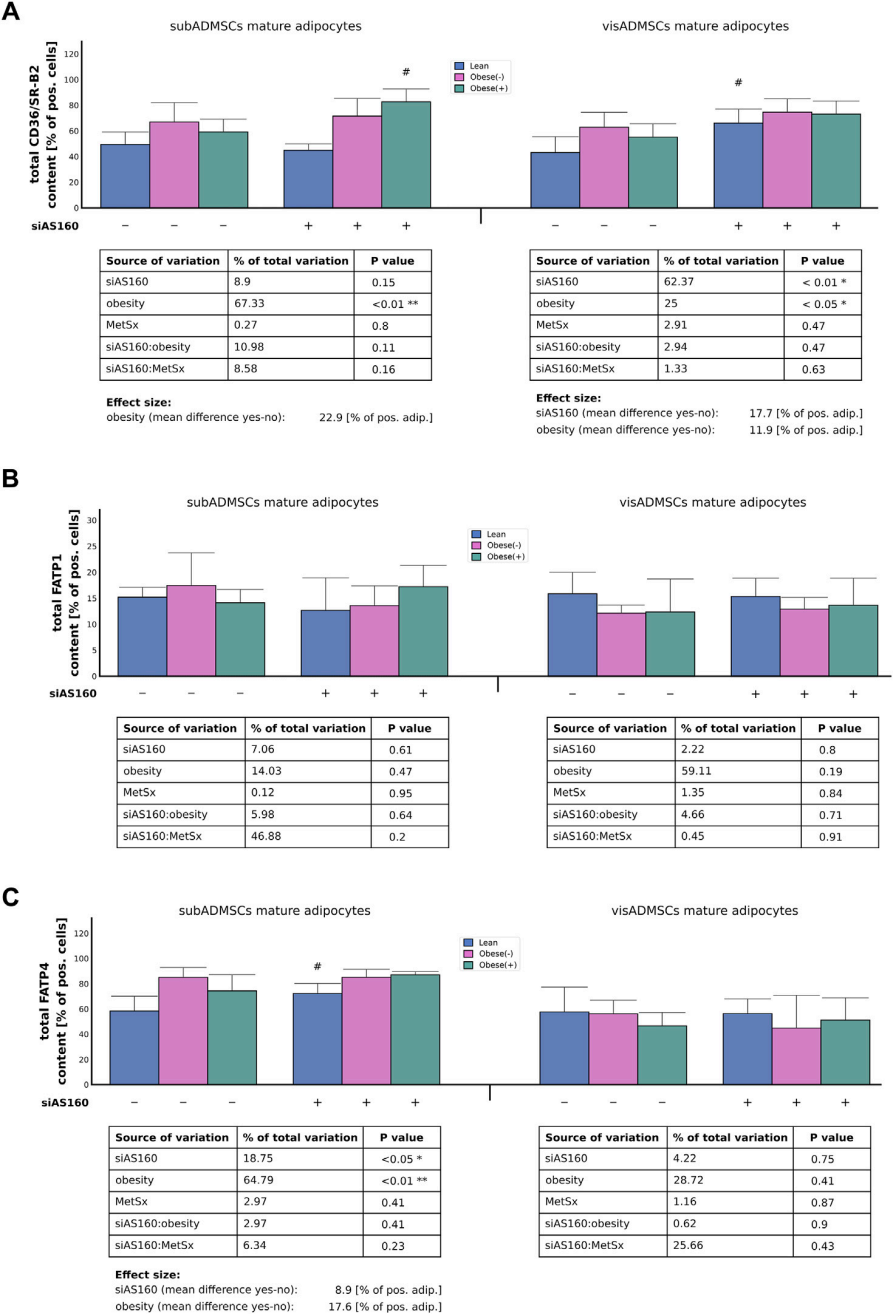
Total protein abundance of fatty acid transporters, i.e., CD36/SR-B2 **(A)**, FATP1 **(B)**, and FATP4 **(C)** in AS160 knocked down mature adipocytes differentiated from ADMSCs of patients with different metabolic status. Data are presented as mean ± SD. # Difference between cells transfected with siRNA targeting AS160 and scrambled siRNA (negative control) within the metabolic status of the donor patient; *p* < 0.05; *n* = 4 for each study group (measurements taken in duplicate). Designations of the groups: NC, negative control containing non-targeting siRNA fragment; siAS160, knockdown of AS160; obese (−), obese without metabolic syndrome; and obese (+), obese with metabolic syndrome.

Although, we could not detect any effect of AS160 silencing on CD36/SR-B2 mRNA expression, the influence of obesity was quite evident (∼55% of data variability). In general, the adipocytes derived from ADMSCs of obese patients had greater expression of CD36/SR-B2 mRNA (∼0.30 [AU], *p* < 0.05, Figure 3A). Similarly, obesity also increased the total CD36/SR-B2 protein content in subADMSCs-derived adipocytes (∼67% of data variability, *p* < 0.05, Figure 4A) and in the mature fat cells of visADMSCs ancestry (25% of data variability, *p* < 0.05, Figure 4A). The condition translated into a greater amount of CD36/SR-B2-positive cells when compared to their lean counterparts. Besides, we noticed that in the case of visceral tissue also, AS160 silencing contributed to a greater CD36/SR-B2 total protein expression [+18% of CD36/SR-B2-positive cells]. On the other hand, none of the examined factors, or their interactions, significantly affected the level of *FATP1* gene and total protein content in the fat cells of ADMSCs ancestry (*p* > 0.05, Figure 3B, Figure 4B). Unlike the aforementioned, obesity significantly increased FATP4 gene expression in the adipocytes derived from subADMSCs (∼76% of data variability, *p* < 0.01, Figure 3C). On average, the cells had a 0.34 [AU] greater expression of the FATP4 transcript than their lean counterparts. Also, in the case of fat cells derived from visADMSCs, obesity influenced FATP4 mRNA expression (∼64% of data variability, *p* < 0.01, Figure 3C). Likewise, the cells had a higher (+0.27 [AU]) expression of the transporter’s mRNA in comparison to their lean analogs. Out of the examined factors, both obesity and AS160 deficiency contributed to changes in the level of total FATP4 protein. Namely, the cells derived from subADMSCs had on average +18% more FATP4-positive cells when they were derived from obese patients. Besides, AS160 silencing increased the number of FATP4-positive cells by 9%. On the contrary, none of the investigated variables seemed to influence the level of total FATP4 protein in the mature fat cells obtained from visADMSCs.

#### 3.3.2 Plasmalemmal expression of fatty acid transport proteins

**FIGURE 5:**
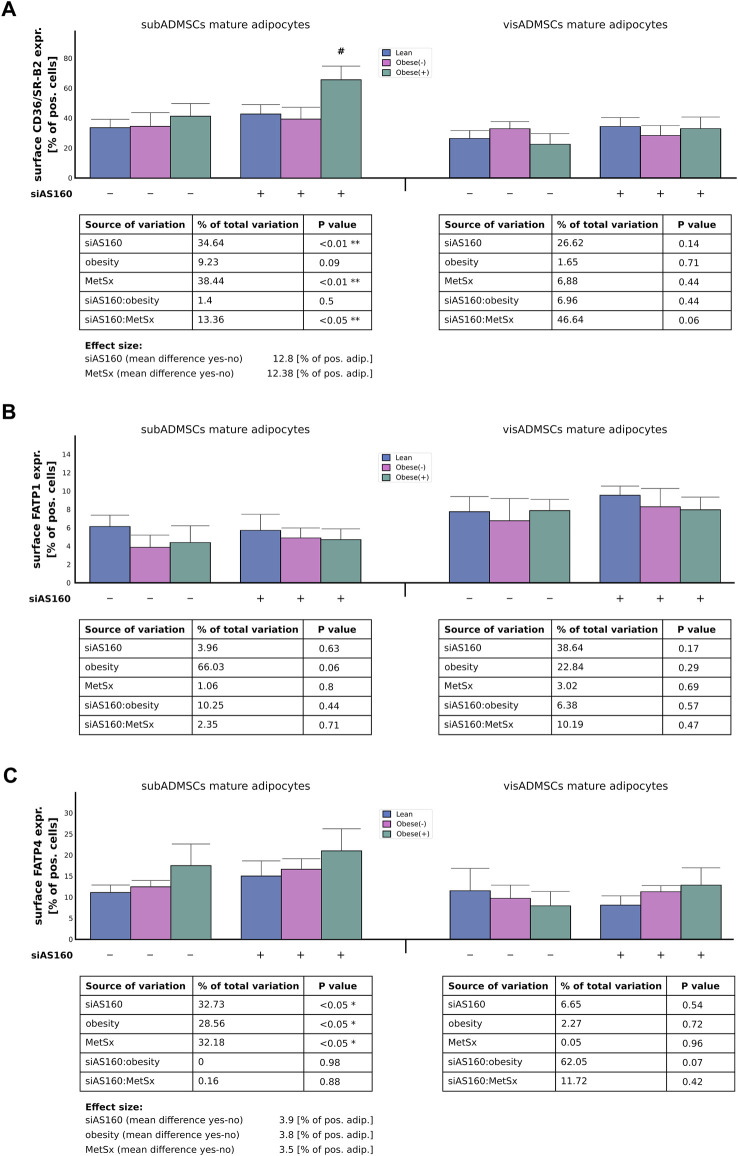
Plasma membrane abundance of fatty acid transporters, i.e., CD36/SR-B2 **(A)**, FATP1 **(B)**, and FATP4 **(C)** in AS160 knocked down mature adipocytes differentiated from ADMSCs of patients with different metabolic status evaluated by flow cytometry analysis. Data are presented as mean ± SD. # Difference between the cells transfected with siRNA targeting AS160 and scrambled siRNA (negative control) within the metabolic status of the donor patient; *p* < 0.05; *n* = 4 for each study group (measurements taken in duplicate). Designations of the groups: NC, negative control containing non-targeting siRNA fragment; siAS160, knockdown of AS160; obese (−), obese without metabolic syndrome; and obese (+), obese with metabolic syndrome.

In the case of the adipocytes derived from subADMSCs, the plasmalemmal expression of CD36/SR-B2 seemed to be affected by both AS160 silencing (35% of data variability, *p* < 0.01) and metabolic syndrome (38% of data variability, *p* < 0.01, Figure 5A). These interventions translated into a 12% increase in the number of adipocytes with the CD36/SR-B2 marker detected by flow cytometry (Figure 5A). Importantly, we observed a positive interaction between the two primary factors, i.e., the cells with both AS160 silencing and metabolic syndrome had even greater surface expression of the examined FA transporter (*p* < 0.05, Figure 5A). Surprisingly, the cells of visADMSCs ancestry seemed to be unaffected by any of the examined factors (or their interactions) with respect to CD36/SR-B2 surface expression. Furthermore, due to high variability in the collected data, we could not confirm the effect of any examined factor on the plasma membrane expression of FATP1 in the adipocytes derived from ADMSCs (*p* > 0.05, Figure 5B). With regard to FATP4, we demonstrated that in the adipocytes obtained from the subADMSCs, three of the examined factors—AS160 silencing (33% of data variability, *p* < 0.05), obesity (29% of data variability, *p* < 0.05), and the presence of metabolic syndrome (32% of data variability, *p* < 0.05)—influenced the plasmalemmal expression of the FATP4 protein (Figure 5C). As evidenced by flow cytometry, all of the aforementioned factors were associated with a greater percentage of FATP4-positive cells (by circa +3–4% of FATP4-positive cells). Surprisingly, the cells that stemmed from visADMSCs seemed to be unaffected by any of the examined factors.

#### 3.3.3 Long-chain fatty acids uptake

**FIGURE 6:**
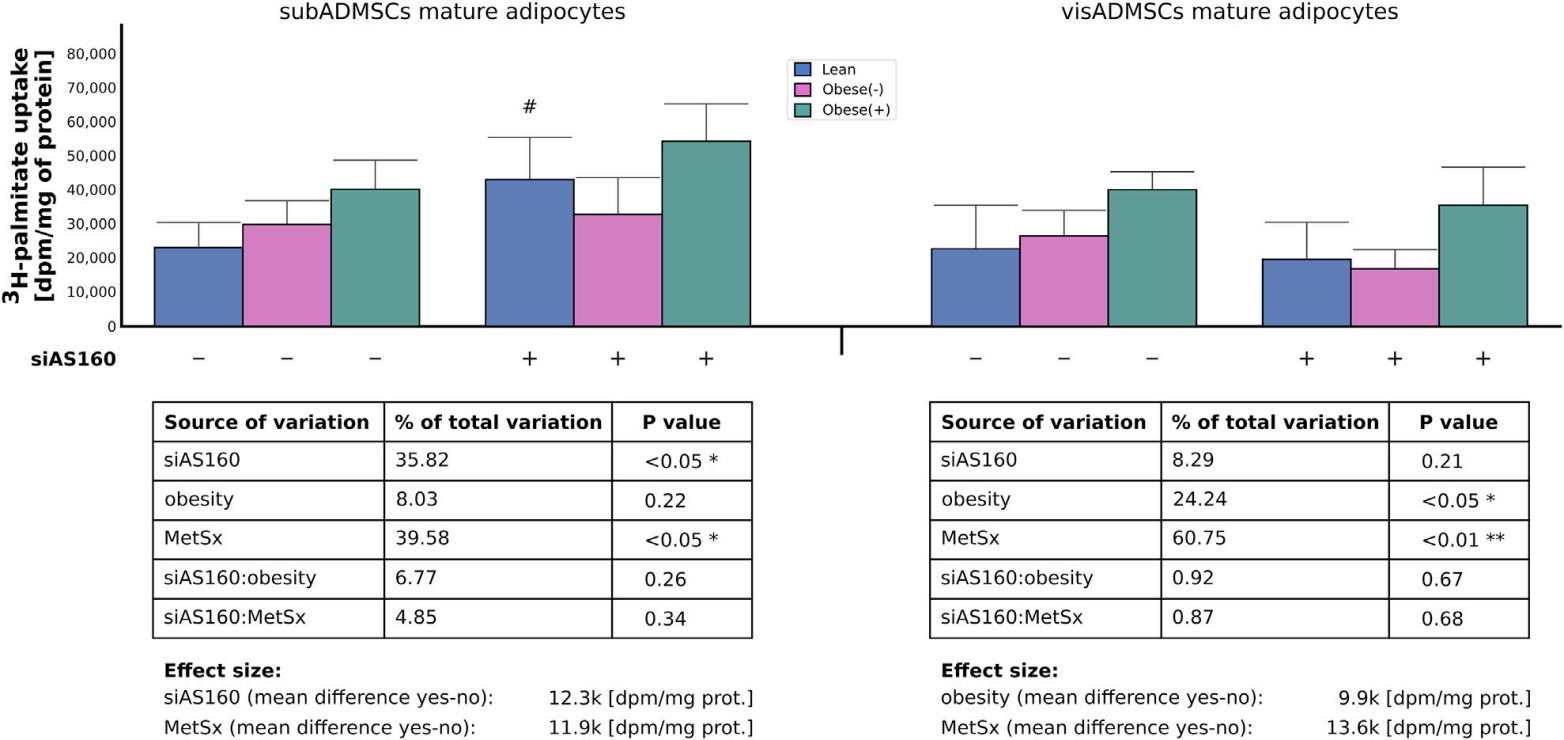
Uptake of ^3^H-palmitate into ADMSCs-derived adipocytes after siRNA-mediated knockdown of AS160. Data are presented as mean ± SD. Values are expressed in dpm per mg of protein. # Difference between the cells transfected with siRNA against AS160 and scrambled siRNA (negative control) within the metabolic status of the donor patient; *p* < 0.05; *n* = 4 for each study group (measurements taken in duplicate). Designations of the groups: NC, negative control containing non-targeting siRNA fragment; siAS160, knockdown of AS160; obese (−), obese without metabolic syndrome; and obese (+), obese with metabolic syndrome.

In line with the aforementioned results, the analyses of the radioactive ^3^H-palmitate uptake in the adipocytes differentiated from subADMSCs demonstrated that the uptake of LCFAs was affected by both AS160 silencing (36% of data variability, *p* < 0.05) and the presence of metabolic syndrome (40% of data variability, *p* < 0.05). Each of the factors contributed to an enhanced influx of the fatty acid by roughly 12,000 [dpm/mg of protein]. In the case of cells of visADMSCs origin, obesity (24% of data variability, *p* < 0.05) and metabolic syndrome (61% of data variability, *p* < 0.01) contributed to a greater uptake of palmitic acid. Obesity increased the uptake by roughly 10,000 (dpm/mg of protein), whereas metabolic syndrome upregulated the palmitate influx by approximately 14,000 (dpm/mg of protein) (Figure 6).

### 3.4 Intracellular lipid content

**FIGURE 7:**
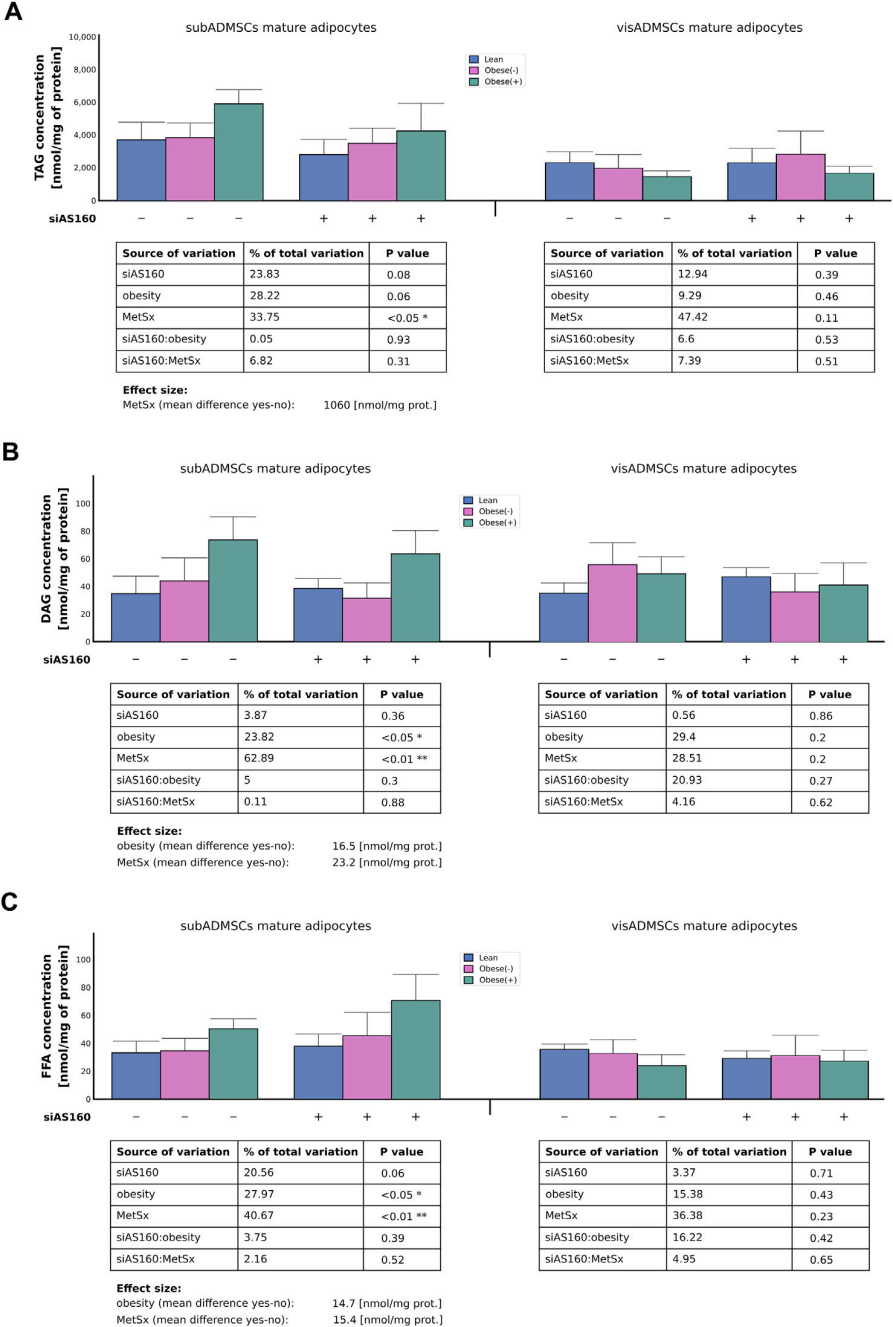
Amount of intracellular lipids, i.e., TAG **(A)**, DAG **(B)**, and FFA **(C)** in AS160 knocked down adipocytes derived from ADMSCs. Data are presented as mean ± SD. Values are expressed in nmol per mg of protein. # Difference between the cells transfected with siRNA targeting AS160 and scrambled siRNA (negative control) within the metabolic status of the donor patient; *p* < 0.05; *n* = 4 for each study group (measurements taken in duplicate). Designations of the groups: NC, negative control containing non-targeting siRNA fragment; siAS160, knockdown of AS160; obese (−), obese without metabolic syndrome; and obese (+), obese with metabolic syndrome.

Gas liquid chromatography revealed that in the mature fat cells obtained from subADMSCs, three factors contributed to the total TAG content, i.e., metabolic syndrome (34% of data variability, *p* < 0.05), obesity (28% of data variability, 0.05 < *p* < 0.1), and AS160 silencing (24% of data variability, 0.05 < *p* < 0.1) (Figure 7A). Only the first aforementioned factor reached the level of statistical significance and caused a greater TAG level within the cells by approximately 1060 [nmol/mg of protein]. On the other hand, in the adipocytes derived from visADMSCs, none of the examined factors significantly influenced the intracellular level of triacylglycerol (*p* > 0.05, Figure 7A). Regarding the intracellular level of total DAG in the fat cells derived from subADMSCs, obesity (24% of data variability, *p* < 0.05) and metabolic syndrome (63% of data variability, *p* < 0.01) affected the lipid level (Figure 7B). The aforementioned factors translated into a greater DAG concentration (by 16 and 23 [nmol/mg of protein], respectively). Interestingly, the total level of DAG in the adipocytes stemming from visADMSCs was relatively stable and showed no systematic alterations (*p* > 0.05, Figure 7B). Furthermore, metabolic syndrome contributed to an increased level (41% of data variability, on average by 15.4 [nmol/mg of protein], *p* < 0.01) of FFA in cells derived from subADMSCs (Figure 7C). Also, obesity was associated with a greater concentration of free fatty acids (*p* < 0.05, Figure 7C) by roughly 14.7 [nmol/mg of protein]. In addition, we did not observe any systematic effect of the examined factors on the total FFA content in the mature fat cells derived from visADMSCs.

### 3.5 Obesity but not AS160 knockdown increased synthesis and degradation of lipids in adipocytes differentiated form ADMSCs of subcutaneous and visceral origins

**FIGURE 8:**
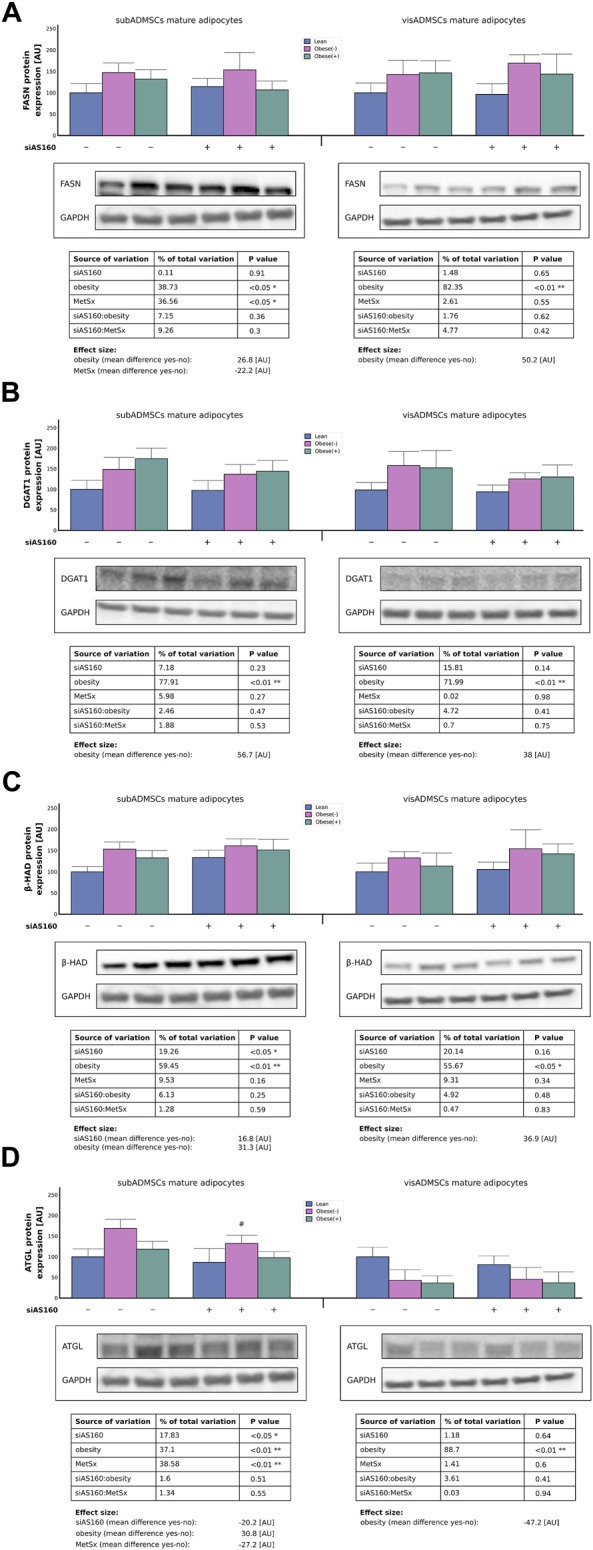
Expression of key enzymes engaged in fatty acid synthesis and utilization, i.e., FASN **(A)**, DGAT1 **(B)**, β-HAD **(C)**, and ATGL **(D)** in AS160 knocked down ADMSCs-derived adipocytes determined by Western blot analysis. Data are presented as mean ± SD. Values are expressed in arbitrary units, i.e., lean non-targeting siRNA group was set at 100 [AU]. # Difference between the cells transfected with siRNA targeting AS160 and scrambled siRNA (negative control) within the metabolic status of the donor patient; *p* < 0.05; *n* = 4 for each study group (measurements taken in duplicate). Representative Western blot images are shown. GAPDH served as the protein loading control. Designations of the groups: NC, negative control containing non-targeting siRNA fragment; siAS160, knockdown of AS160; obese (−), obese without metabolic syndrome; and obese (+), obese with metabolic syndrome.

Both obesity (39% of data variability, *p* < 0.05) and metabolic syndrome (37% of data variability, *p* < 0.05) affected the expression of FASN protein in the adipocytes that were obtained from subADMSCs (Figure 8A). Interestingly, the cells from obese groups had a greater (+27 [AU]) expression of FASN protein, whereas those from the group with metabolic syndrome were characterized by a lower level (−22 [AU]) of the enzyme (Figure 8A). With respect to the adipocytes of visceral provenance, obesity was the only factor that contributed to the augmented level [+50 (AU)] of FASN protein (82% of data variability, *p* < 0.05, Figure 8A). Furthermore, the Western blot analysis revealed that obesity contributed (78% and 72% of data variability, *p* < 0.01, for the cells of subADMSCs and visADMSCs ancestry, respectively) to the increased level of DGAT1 protein (+57 [AU] in subADMSCs and +38 [AU] in visADMSCs, *p* < 0.01, Figure 8B). With regard to the β-HAD protein level, we found that it was dependent on both AS160 silencing (19% of data variability for the adipocytes derived from subADMSCs, *p* < 0.05) and obesity (∼59% and 56% of data variability, *p* < 0.01 and *p* < 0.05, for cells of subADMSCs and visADMSCs provenance, respectively). The former was associated with approximately 17 [AU] greater expression of the β-HAD protein. The latter condition contributed to an increased level of the enzyme by +31 [AU] in subADMSCs and +37 [AU] in visADMSCs (Figure 8C). Additionally, our analysis showed that three of the examined factors, i.e., metabolic syndrome, obesity, and AS160 silencing, affected the expression of the ATGL protein in subADMSCs-derived adipocytes (39%, 37%, and 18% of data variability, respectively). Obesity translated into a greater expression of the enzyme (+31 [AU], *p* < 0.01, Figure 8D). On the other hand, both gene silencing and metabolic syndrome resulted in a smaller amount of ATGL protein (−20 [AU] and −27 [AU], respectively). In the adipocytes stemming from visADMSCs, only obesity appeared to affect the expression of the enzyme (89% of data variability, *p* < 0.01, Figure 8D). Here, the cells obtained from obese patients had a lower expression of ATGL by roughly 47 [AU].

## 4 Discussion

AS160 is a key regulator of glucose metabolism and signaling, known for facilitating the cellular glucose uptake in skeletal muscles and adipocytes. Recent studies underlying AS160 functioning have pointed toward a prominent and pivotal role of the protein in various steps of lipid metabolism. It was shown that a deficiency in the RabGAP activator (AS160) led to alterations in lipid composition in myocytes and to increased palmitic acid oxidation in both isolated skeletal muscle and cultured muscle cells (Chadt et al., 2015; Mikłosz et al., 2017). Furthermore, AS160 silencing increased total and surface expression of CD36/SR-B2 in L6 myotubes (Mikłosz et al., 2017) and HL-1 cardiomyocytes (Samovski et al., 2012), respectively. Similarly, SLC27A4/FATP4 protein abundance and subcellular localization in skeletal muscle cells were upregulated in AS160 knockdown mice (Benninghoff et al., 2020). Finally, a shortage of AS160 (TBC1D4) resulted in a significantly increased LCFA uptake in the cultured adipocytes differentiated from adipose-derived mesenchymal stem cells of metabolically healthy lean women (Mikłosz et al., 2021). Encouraged by the results of our investigations, here, we further explore the modulatory role of AS160 on the lipid profile in the adipocytes derived from ADMSCs of patients with morbid obesity alone or with coexisting metabolic syndrome. To verify this, we knocked down AS160 (reduced expression of the gene’s mRNA by approximately 70%–80% and its protein by roughly 45%–80%) in adipocytes derived from patients with different metabolic status. Interestingly, we noticed that the level of AS160 mRNA/protein was significantly greater in the cells stemming from patients with obesity (by roughly 30%–40%). It is hard to satisfactorily explain this finding since reports on the total AS160 level in obesity seem to be missing. Still, an increased content of AS160 in the mature human adipocytes should act as a negative regulator of the energy substrate uptake (Sano et al., 2007). If so, the increased amount of AS160 in the adipocytes originated from obese patients may curtail the intracellular influx of fatty acids and thus reduce the risk of cellular lipid overload observed in this condition. This is in agreement with our former study, in which we suggested that this could be a compensatory mechanism present to counterbalance the overexpression of total FA transporters (e.g., total protein content of CD36/SR-B2) by reducing their translocation to the plasma membrane (Mikłosz et al., 2022a). If that is the case, then one would expect AS160 silencing to exert a pronounced effect on FA transporters' surface expression and cellular FFA uptake. Surprisingly, we did not observe such dramatic changes in the AS160-deficient cells from the “obese” groups. Granted, in the adipocytes of subcutaneous origin, we noticed an interaction between the two examined factors (MetSx and siAS160) with respect to the surface amount of CD36/SR-B2. This indicates that the amount of FA transporter was greater in this group than that expected based on the added effects of MetSx and gene silencing alone. Still, we were unable to confirm the existence of any interaction between the factors (silencing and obesity or metabolic syndrome) with respect to palmitic acid uptake. Therefore, it seems that the precise role of AS160 in obesity requires further studies.

Under basal conditions, AS160 exerts an inhibitory effect on the small Rab GTPases, thus attenuating the translocation of FA transporter–containing vesicles to the plasma membrane. Consistent with the role of AS160 in retaining fatty acid transporters, we showed that AS160 deficiency increases surface expression of CD36/SR-B2 and SLC27A4/FATP4 in mature adipocytes of subADMSCs origin. Similarly, it was shown that the knockdown of AS160 by the siRNA strategy in rat L6-myotubes, mouse HL-1 cardiomyocytes or human ADMSCs-derived adipocytes of lean subjects markedly increased the rate of long-chain fatty acid transport driven mostly by the redistribution of CD36/SR-B2 and/or SLC27A4/FATP4 from the intracellular storage depot to the plasma membrane (Samovski et al., 2012; Mikłosz et al., 2017; Benninghoff et al., 2020; Mikłosz et al., 2021) Furthermore, although the transcript level of FA transporters seemed to be virtually unaffected by AS160 silencing, we observed an increase in total abundance of CD36/SR-B2 in visADMSCs-derived adipocytes and SLC27A4/FATP4 in the adipocytes of subADMSCs provenance. Although, the majority of the literature indicates that AS160 is mainly involved in cellular trafficking (i.e., changing the cellular location of transport proteins and not their total amount), this protein also interacts with a lot of Rabs (e.g., Rab28), i.e., molecules regulating sorting and lysosomal degradation, and as such may potentially contribute to the changes in the total level of certain proteins (Chadt et al., 2015). Nevertheless, our intervention (i.e., AS160 gene silencing) enhanced the cellular influx of palmitic acid in the cells of subADMSCs' ancestry. Overall, the modulation of AS160 expression exerted the effects virtually only on adipocytes derived from subADMSCs. The observed response pattern with regard to subADMSCs- and visADMSCs-derived cells is puzzling. In general, visceral adipose tissue expansion is believed to be a major predictive factor in the development of metabolic abnormalities. On the other hand, excessive lipid storage in subcutaneous depots is considered to be relatively safe. For instance, according to the overflow hypothesis (Pandžić Jakšić and Grizelj, 2016), SAT is the first to expand due to chronic energy surplus, and only when its buffering capacity exceeds, fatty acids overflow toward VAT and other metabolically adverse ectopic depots. The aforementioned would confer a physiological rationale on the observation that AS160 (a key cellular regulator of fatty acids trafficking) appears to mostly affect the adipocytes of subADMSC origin. There is yet another possible explanation of the observed phenomena. Namely, some authors believe that ADMSCs are early sensors of metabolic alterations in the body (Serena et al., 2016; Lefevre et al., 2021). We must bear in mind that obesity-induced inflammation, which promotes metabolic syndrome, originates and progresses mostly in visceral fat (Serena et al., 2016). In view of the aforementioned, ADMSCs isolated from the visceral region of obese patients would be expected to display more adipogenic and immune dysfunctions than the progenitor cells obtained from subcutaneous localization. The aforementioned may impair their regulatory functions, which may be the reason for the limited effects of AS160 knockdown observed in these cells.

Growing evidence suggests that functional differences in adipose tissue depots and the impact of their dysfunction on the overall body’s metabolism may be based on the intrinsic differences in the tissue’s progenitor stem cells (Baglioni et al., 2012; Ong et al., 2014). The greater ability of subADMSCs to proliferate and differentiate into adipocytes than the ADMSCs of visceral provenance may support the hypothesis of adipose tissue expansion underlying the pathogenesis of metabolic diseases (Kim et al., 2016). The presence of hypertrophic adipocytes is a key feature of impaired adipose tissue function, and enlarged adipocytes have an impaired ability to store extra energy. Recently, we have shown that obesity significantly increases cell volume of human adipocytes derived exclusively from visADMSCs and also decreases their capacity to take up FA surplus when compared with smaller size subADMSCs-derived adipocytes (Mikłosz et al., 2022a). Upon chronic fatty acid oversupply, increased fatty acid influx is accompanied by progressive lipid storage. Herein, we found that obesity significantly increased lipid content (FFA, DAG, and TAG) but solely in the adipocytes of subcutaneous (subADMSCs) origin. Moreover, the effect was exacerbated by the onset of metabolic syndrome. The aforementioned suggests that subADMSC adipocytes did not exceed their lipid storage capacity and could still prevent lipid overload in visADMSC-derived adipocytes.

Lipid accumulation results from several mechanisms such as inflated FA uptake and storage as TAG in lipid droplets, greater fatty acid *de novo* synthesis, and decreased lipolysis in adipose tissue (Roberts et al., 2009). An increased influx of LCFAs may therefore result from a greater availability of FA transporters (CD36/SR-B2 and FATP4) but can also be driven by increased lipid catabolism in mature adipocytes differentiated from ADMSCs. Importantly, obesity enhances the expression of the key enzymes responsible for fatty acid synthesis (FASN), triglyceride synthesis (DGAT1), and lipid oxidation (β-HAD) in both subADMSCs- and visADMSCs-derived adipocytes. Accordingly, Berndt et al. (2007) had found increased FASN expression in the adipose tissue of obese individuals, which was positively correlated with adipocyte size (Berndt et al., 2007). Among the known regulators of FASN expression are hyperinsulinemia, elevated serum triacylglycerols, and NEFAs, all of which are increased in obesity and thus result in augmented lipogenesis. The upregulation of the lipogenic marker provides substrates for triglyceride synthesis, which subsequently enhances fat deposition in adipocytes (Chitraju et al., 2017). On the other hand, excessive fatty acid supply increases the oxidative capacity of the mitochondria, as evidenced by increased β-HAD expression. However, when the capacity of mitochondrial oxidation exceeds, lipids are stored as TAGs and might be converted into lipid metabolites such as DAG, which is known to inhibit insulin signaling, thus causing insulin resistance. Moreover, obesity markedly reduces ATGL expression in visADMSCs-derived adipocytes, which is partially consistent with the results of the study by Yao-Borengasser et al. (2011). The authors have reported reduced ATGL protein expression in the visceral adipose tissue of obese and insulin-resistant individuals when compared with their lean counterparts, although there was no change in ATGL mRNA levels (Yao-Borengasser et al., 2011). In general, regardless of the metabolic status of humans, subcutaneous adipocytes have greater abundance of ATGL lipase, indicating their higher rate of lipid turnover. During lipolysis, adipose tissue FAs are liberated from TAG, but a large fraction of these FAs (around 60%–70%) is re-esterified back to TAG catalyzed by DGAT1 (Chitraju et al., 2017). Accordingly, we found that the protein level of DGAT1 was upregulated in mature adipocytes differentiated form ADMSCs of obese individuals. This is in line with previous reports that have generally suggested that DGAT1 levels increase during obesity, and its function is important to prevent excessive accumulation of ectopic lipids but not so much to preserve fat mass. Chitraju et al. (2017) indicated that DGAT1-mediated re-esterification protects adipocytes from endoplasmic reticulum stress, allowing organisms to adapt to constantly changing energy conditions (Chitraju et al., 2017).

In conclusion, subADMSCs-derived adipocytes better reflect the metabolic status of donor patients, as obesity and metabolic syndrome significantly increased their lipid content when compared to visADMSCs-derived adipocytes. These differential responses of *in vitro*–cultured adipocytes to the metabolic status of donor patients suggest that subADMSCs are more susceptible to obesity-related metabolic reprogramming. Furthermore, our data demonstrate that in human adipocytes, AS160 controls LCFA entry via CD36/SR-B2 and/or SLC27A4/FATP4 transporters. However, AS160 does not appear to significantly affect the phenotype of adipocytes stemming from obese patients with respect to their cellular lipid content (TAG, DAG, and FFA), possibly due to the concomitant higher potential of the adipocytes for lipid turnover and utilization.

## Figures and Tables

**Table:** 

**ADMSCs**	Adipose-derived mesenchymal stem cells
**ALT**	Alanine transaminase
**AST**	Aspartate transaminase
**AS160/TBC1D4**	Akt substrate of 160 kDa (AS160), also known as TBC1D4
**ATGL**	Adipose triglyceride lipase
**BMI**	Body mass index
**CRP**	C-reactive protein
**β-HAD**	Beta-hydroxyacyl CoA dehydrogenase
**CD36-SR-B2**	Cluster of differentiation 36 (CD36), also known as scavenger receptor class B
**DAG**	Diacylglycerol
**DGAT1**	Diacylglycerol acyltransferase 1
**DP**	Diastolic pressure
**FAs**	Fatty acids
**FASN**	Fatty acid synthase
**FFA**	Free fatty acids
**GAPDH**	Glyceraldehyde-3-phosphate dehydrogenase
**GLUT 4**	Glucose transporter 4
**HDL**	High-density lipoprotein
**HGB**	Hemoglobin
**HOMA-IR**	Homeostatic model assessment of insulin resistance
**INR**	International normalized ratio
**LCFAs**	Long-chain fatty acids
**LDL**	Low-density lipoprotein
**MetSx**	Metabolic syndrome
**NC**	Negative control containing non-targeting siRNA fragment
**Obese(−)**	Obese without metabolic syndrome
**Obese(+)**	Obese with metabolic syndrome
**PLT**	Platelet count
**RabGAP**	Rab GTPase–activating protein
**RBC**	Red blood cell count
**SAT**	Subcutaneous adipose tissue
**siAS160**	Knockdown of AS160
**SLC27A1/FATP1**	Member of the SLC27 family of fatty acid transport protein 1
**SLC27A4/FATP4**	Member of the SLC27 family of fatty acid transport protein 4
**SP**	Systolic pressure
**subADMSCs**	ADMSCs derived from subcutaneous adipose tissue
**SVF**	Stromal vascular fraction
**TAG**	Triacylglycerol
**TBC1D1**	TBC1 domain family member 1
**TBC1D4**	TBC1 domain family member 4
**VAT**	Visceral adipose tissue
**visADMSCs**	ADMSCs derived from visceral adipose tissue
**WAT**	White adipose tissue
**WBC**	White blood cell count
**WHR**	Waist–hip ratio

## Data Availability

The datasets presented in this study can be found in online repositories. The names of the repository/repositories and accession number(s) can be found at: https://ppm.umb.edu.pl/info/researchdata/UMB4d427c3c3d20496493b0631a3f96a3c5/.

## References

[B1] Aguer C. Foretz M. Lantier L. Hebrard S. Viollet B. Mercier J. (2011). Increased FAT/CD36 cycling and lipid accumulation in myotubes derived from obese type 2 diabetic patients. PLoS One 6. 10.1371/journal.pone.0028981 PMC3241688 22194967

[B2] Badimon L. Cubedo J. (2017). Adipose tissue depots and inflammation: Effects on plasticity and resident mesenchymal stem cell function. Cardiovasc. Res. 113, 1064–1073. 10.1093/cvr/cvx096 28498891

[B3] Baglioni S. Cantini G. Poli G. Francalanci M. Squecco R. Franco A. (2012). Functional differences in visceral and subcutaneous fat pads originate from differences in the adipose stem cell. PLoS One 7, e36569. 10.1371/journal.pone.0036569 22574183 PMC3344924

[B4] Benninghoff T. Espelage L. Eickelschulte S. Zeinert I. Sinowenka I. Müller F. (2020). The rabgaps tbc1d1 and tbc1d4 control uptake of long-chain fatty acids into skeletal muscle via fatty acid transporter SLC27A4/FATP4. Diabetes 69, 2281–2293. 10.2337/db20-0180 32868338

[B5] Berndt J. Kovacs P. Ruschke K. Klöting N. Fasshauer M. Schön M. R. (2007). Fatty acid synthase gene expression in human adipose tissue: Association with obesity and type 2 diabetes. Diabetologia 50, 1472–1480. 10.1007/s00125-007-0689-x 17492427

[B6] Cao M. Pan Q. Dong H. Yuan X. Li Y. Sun Z. (2015). Adipose-derived mesenchymal stem cells improve glucose homeostasis in high-fat diet-induced obese mice. Stem Cell Res. Ther. 6, 1–13. 10.1186/s13287-015-0201-3 26519255 PMC4628312

[B7] Chadt A. Immisch A. De Wendt C. Springer C. Zhou Z. Stermann T. (2015). Deletion of both rab-GTPase-activating proteins TBC1D1 and TBC1D4 in mice eliminates insulin- and AICAR-stimulated glucose transport. Diabetes 64, 746–759. 10.2337/db14-0368 25249576

[B8] Chavez J. A. Summers S. A. (2003). Characterizing the effects of saturated fatty acids on insulin signaling and ceramide and diacylglycerol accumulation in 3T3-L1 adipocytes and C2C12 myotubes. Arch. Biochem. Biophys. 419, 101–109. 10.1016/j.abb.2003.08.020 14592453

[B9] Chitraju C. Mejhert N. Haas J. T. Diaz-Ramirez L. G. Grueter C. A. Imbriglio J. E. (2017). Triglyceride synthesis by DGAT1 protects adipocytes from lipid-induced ER stress during lipolysis. Cell Metab. 26, 407–418. 10.1016/j.cmet.2017.07.012 28768178 PMC6195226

[B10] Coburn C. T. Knapp J. Febbraio M. Beets A. L. Silverstein R. L. Abumrad N. A. (2000). Defective uptake and utilization of long chain fatty acids in muscle and adipose tissues of CD36 knockout mice. J. Biol. Chem. 275, 32523–32529. 10.1074/jbc.M003826200 10913136

[B11] Dominici M. Le Blanc K. Mueller I. Slaper-Cortenbach I. Marini F. C. Krause D. S. (2006). Minimal criteria for defining multipotent mesenchymal stromal cells. The International Society for Cellular Therapy position statement. Cytotherapy 8, 315–317. 10.1080/14653240600855905 16923606

[B12] Hames K. C. Vella A. Kemp B. J. Jensen M. D. (2014). Free fatty acid uptake in humans with CD36 deficiency. Diabetes 63, 3606–3614. 10.2337/db14-0369 24917573 PMC4207394

[B13] Hao J. W. Wang J. Guo H. Zhao Y. Y. Sun H. H. Li Y. F. (2020). CD36 facilitates fatty acid uptake by dynamic palmitoylation-regulated endocytosis. Nat. Commun. 11, 1–16. 10.1038/s41467-020-18565-8 32958780 PMC7505845

[B14] Hwang I. Kim J. B. (2019). Two faces of white adipose tissue with heterogeneous adipogenic progenitors. Diabetes Metab. J. 43, 752–762. 10.4093/dmj.2019.0174 31902145 PMC6943255

[B15] Ishida M. Tatsumi K. Okumoto K. Kaji H. (2020). Adipose tissue-derived stem cell sheet improves glucose metabolism in obese mice. Stem Cells Dev. 29, 488–497. 10.1089/scd.2019.0250 32075539

[B16] Jaber H. Issa K. Eid A. Saleh F. A. (2021). The therapeutic effects of adipose-derived mesenchymal stem cells on obesity and its associated diseases in diet-induced obese mice. Sci. Rep. 11, 1–9. 10.1038/s41598-021-85917-9 33737713 PMC7973738

[B17] Kim B. Lee B. Kim M. K. Gong S. P. Park N. H. Chung H. H. (2016). Gene expression profiles of human subcutaneous and visceral adipose-derived stem cells. Cell biochem. Funct. 34, 563–571. 10.1002/cbf.3228 27859461

[B18] Lee M. J. Wu Y. Fried S. K. (2013). Adipose tissue heterogeneity: Implication of depot differences in adipose tissue for obesity complications. Mol. Asp. Med. 34, 1–11. 10.1016/j.mam.2012.10.001 PMC3549425 23068073

[B19] Lefevre C. Chartoire D. Ferraz J. C. Verdier T. Pinteur C. Chanon S. (2021). Obesity activates immunomodulating properties of mesenchymal stem cells in adipose tissue with differences between localizations. FASEB J. 35, 1–18. 10.1096/fj.202002046RR 33993539

[B20] Liao N. Pan F. Wang Y. Zheng Y. Xu B. Chen W. (2016). Adipose tissue-derived stem cells promote the reversion of non-alcoholic fatty liver disease: An *in vivo* study. Int. J. Mol. Med. 37, 1389–1396. 10.3892/ijmm.2016.2528 26986083

[B21] Mafakheri S. Chadt A. Al-Hasani H. (2018). Regulation of RabGAPs involved in insulin action. Biochem. Soc. Trans. 46, 683–690. 10.1042/BST20170479 29784647

[B22] MayoClinic (2023). Metabolic syndrome. Available at:https://www.mayoclinic.org/diseases-conditions/metabolic-syndrome/diagnosis-treatment/drc-20351921 (Accessed July 4, 2023).

[B23] Mikłosz A. Chabowski A. (2023). Adipose-derived mesenchymal stem cells therapy as a new treatment option for diabetes mellitus. J. Clin. Endocrinol. Metab. 14, dgad142. 10.1210/clinem/dgad142 PMC10348459 36916961

[B24] Mikłosz A. Łukaszuk B. Żendzian-Piotrowska M. Brańska-Januszewska J. Ostrowska H. Chabowski A. (2017). Challenging of as160/tbc1d4 alters intracellular lipid milieu in L6 myotubes incubated with palmitate. J. Cell. Physiol. 232, 2373–2386. 10.1002/jcp.25632 27714805 PMC5485047

[B25] Mikłosz A. Łukaszuk B. Supruniuk E. Grubczak K. Moniuszko M. Choromańska B. (2021). Does TBC1D4 (AS160) or TBC1D1 deficiency affect the expression of fatty acid handling proteins in the adipocytes differentiated from human adipose-derived mesenchymal stem cells (ADMSCs) obtained from subcutaneous and visceral fat depots? Cells 10 (6), 1515. 10.3390/cells10061515 34208471 PMC8235367

[B26] Mikłosz A. Łukaszuk B. Supruniuk E. Grubczak K. Starosz A. Kusaczuk M. (2022a). The phenotype of the adipocytes derived from subcutaneous and visceral ADMSCs is altered when they originate from morbidly obese women: Is there a memory effect? Cells 11 (9), 1435. 10.3390/cells11091435 35563741 PMC9099624

[B27] Mikłosz A. Łukaszuk B. Zendzian-Piotrowska M. Kurek K. Chabowski A. (2016). The effects of AS160 modulation on fatty acid transporters expression and lipid profile in L6 myotubes. Cell. Physiol. biochem. 38, 267–282. 10.1159/000438628 26784579

[B28] Mikłosz A. Nikitiuk B. E. Chabowski A. (2022b). Using adipose-derived mesenchymal stem cells to fight the metabolic complications of obesity: Where do we stand? Obes. Rev. 23 (5), e13413. 10.1111/obr.13413 34985174 PMC9285813

[B29] Ong W. K. Tan C. S. Chan K. L. Goesantoso G. G. Chan X. H. D. Chan E. (2014). Identification of specific cell-surface markers of adipose-derived stem cells from subcutaneous and visceral fat depots. Stem Cell Rep. 2, 171–179. 10.1016/j.stemcr.2014.01.002 PMC3923222 24527391

[B30] Pandžić Jakšić V. Grizelj D. (2016). Under the surface of subcutaneous adipose tissue biology. Acta Dermatovenerol Croat. 24, 250–260. 28128075

[B31] Peinado J. R. Jimenez-Gomez Y. Pulido M. R. Ortega-Bellido M. Diaz-Lopez C. Padillo F. J. (2010). The stromal-vascular fraction of adipose tissue contributes to major differences between subcutaneous and visceral fat depots. Proteomics 10, 3356–3366. 10.1002/pmic.201000350 20706982

[B32] Pepino M. Y. Kuda O. Samovski D. Abumrad N. A. (2014). Structure-function of CD36 and importance of fatty acid signal transduction in fat metabolism. Annu. Rev. Nutr. 34, 281–303. 10.1146/annurev-nutr-071812-161220 24850384 PMC4329921

[B33] Pfaffl M. W. (2001). A new mathematical model for relative quantification in real-time RT-PCR. Nucleic Acids Res. 29 (9), e45. 10.1093/nar/29.9.e45 11328886 PMC55695

[B34] Reyes-Farias M. Fos-Domenech J. Serra D. Herrero L. Sánchez-Infantes D. (2021). White adipose tissue dysfunction in obesity and aging. Biochem. Pharmacol. 192, 114723. 10.1016/j.bcp.2021.114723 34364887

[B35] Roberts R. Hodson L. Dennis A. L. Neville M. J. Humphreys S. M. Harnden K. E. (2009). Markers of de novo lipogenesis in adipose tissue: Associations with small adipocytes and insulin sensitivity in humans. Diabetologia 52, 882–890. 10.1007/s00125-009-1300-4 19252892

[B36] Sakamoto K. Holman G. D. (2008). Emerging role for AS160/TBC1D4 and TBC1D1 in the regulation of GLUT4 traffic. Am. J. Physiol. - Endocrinol. Metab. 295, 29–37. 10.1152/ajpendo.90331.2008 PMC2493596 18477703

[B37] Samovski D. Su X. Xu Y. Abumrad N. A. Stahl P. D. (2012). Insulin and AMPK regulate FA translocase/CD36 plasma membrane recruitment in cardiomyocytes via Rab GAP AS160 and Rab8a Rab GTPase. J. Lipid Res. 53, 709–717. 10.1194/jlr.M023424 22315395 PMC3307647

[B38] Sano H. Eguez L. Teruel M. N. Fukuda M. Chuang T. D. Chavez J. A. (2007). Rab10, a target of the AS160 rab GAP, is required for insulin-stimulated translocation of GLUT4 to the adipocyte plasma membrane. Cell Metab. 5, 293–303. 10.1016/j.cmet.2007.03.001 17403373

[B39] Serena C. Keiran N. Ceperuelo-Mallafre V. Ejarque M. Fradera R. Roche K. (2016). Obesity and type 2 diabetes alters the immune properties of human adipose derived stem cells. Stem Cells 34, 2559–2573. 10.1002/stem.2429 27352919

[B40] Silva K. R. Liechocki S. Carneiro J. R. Claudio-Da-Silva C. Maya-Monteiro C. M. Borojevic R. (2015). Stromal-vascular fraction content and adipose stem cell behavior are altered in morbid obese and post bariatric surgery ex-obese women. Stem Cell Res. Ther. 6, 1–13. 10.1186/s13287-015-0029-x 25884374 PMC4435525

[B41] Stafeev I. S. Sklyanik I. A. Yah’yaev K. A. Shestakova E. A. Yurasov A. V. Karmadonov A. V. (2019). Low AS160 and high SGK basal phosphorylation associates with impaired incretin profile and type 2 diabetes in adipose tissue of obese patients. Diabetes Res. Clin. Pract. 158, 107928. 10.1016/j.diabres.2019.107928 31734225

[B42] Wu L. Xu D. Zhou L. Xie B. Yu L. Yang H. (2014). Rab8a-AS160-MSS4 regulatory circuit controls lipid droplet fusion and growth. Dev. Cell 30, 378–393. 10.1016/j.devcel.2014.07.005 25158853

[B43] Yao-Borengasser A. Varma V. Coker R. H. Ranganathan G. Phanavanh B. Rasouli N. (2011). Adipose triglyceride lipase expression in human adipose tissue and muscle. Role in insulin resistance and response to training and pioglitazone. Metabolism 60, 1012–1020. 10.1016/j.metabol.2010.10.005 21129760 PMC3062961

