# Supplementary material for: RabGAP AS160/TBC1D4 deficiency increases long-chain fatty acid transport but has little additional effect on obesity and metabolic syndrome in ADMSCs-derived adipocytes of morbidly obese women
Source: Front Mol Biosci. 2023 Aug 3;10:1232159. doi: 10.3389/fmolb.2023.1232159 (PMC10435366; doi:10.3389/fmolb.2023.1232159)
Supplement: Supplementary file 1 [file Table1.DOCX]

**Table 1.** Clinical characteristics of the lean and morbidly obese women ( adipose tissue donors).

|  | **Lean** | **Obese(-)** | **Obese(+)** |
| --- | --- | --- | --- |
| **Age [yrs]** | 61.0 ± 3.367 | 61.25 ± 2.63 | 62.0 ± 3.162 |
| **BMI [kg/m^2^]** | 23.33 ± 2.043 | 42.43 ± 2.243* | 50.12 ± 4.511*^ |
| **WHR** | 0.84 ± 0.143 | 0.92 ± 0.029 | 0.92 ± 0.025 |
| **Glucose [mg]dL]** | 79.75 ± 10.243 | 96.75 ± 12.894 | 105.0 ± 4.83* |
| **Insulin** | 6.99 ± 0.672 | 19.88 ± 2.622* | 27.5 ± 7.148* |
| **HOMA-IR** | 1.36 ± 0.076 | 4.74 ± 0.787* | 7.17 ± 2.108* |
| **CRP [mg/L]** | 5.78 ± 0.435 | 9.49 ± 0.848* | 12.58 ± 2.457* |
| **Systolic Pressure**  **[mmHg]** | 113.75 ± 4.787 | 118.75 ± 11.087 | 153.75 ± 9.465* |
| **Diastolic Pressure [mmHg]** | 77.5 ± 5.0 | 86.25 ± 4.787 | 92.5 ± 5.0* |
| **ALT [IU/L]** | 26.5 ± 4.041 | 38.75 ± 6.344* | 31.25 ± 1.893 |
| **AST [IU/L]** | 21.0 ± 2.0 | 18.5 ± 3.697 | 32.25 ± 8.221^ |
| **Cholesterol [mg/dL]** | 162.75 ± 6.702 | 163.25 ± 11.529 | 199.25 ± 57.996 |
| **HDL [mg/dL]** | 59.25 ± 6.238 | 49.25 ± 4.272* | 37.0 ± 12.41* |
| **LDL [mg/dL]** | 107.75 ± 1.5 | 116.5 ± 9.147 | 133.25 ± 35.929 |
| **TAG [mg/dL]** | 142.5 ± 4.655 | 149.5 ± 6.455 | 175.0 ± 19.201* |
| **RBC [mln/mm^3^]** | 4.77 ± 0.149 | 4.62 ± 0.405 | 4.91 ± 0.285 |
| **Hb [g/dL]** | 13.62 ± 1.081 | 13.77 ± 0.892 | 12.88 ± 0.793 |
| **WBC [thous./mm^3^]** | 8.08 ± 1.962 | 7.44 ± 0.668 | 7.63 ± 1.666 |
| **Platelets [thous./mm^3^]** | 294.75 ± 23.128 | 275.25 ± 50.129 | 273.5 ± 11.902 |

Data are presented as mean and standard deviation. p < 0.05; * – indicates significant differences vs. lean patients; ^ – indicates significant differences vs. Obese(-) group.

ALT – alanine transaminase; AST – aspartate transaminase; BMI – body mass index; CRP – C-reactive protein; DP – diastolic pressure; HDL – high-density lipoprotein; HGB – hemoglobin; HOMA-IR – homeostatic model assessment of insulin resistance; INR – international normalized ratio; LDL – low-density lipoprotein; PLT – platelet count; RBC – red blood cell count; SP – systolic pressure; TAG – triacylgycerol; WBC – white blood cell count; WHR – waist-hip ratio
