# Supplementary material for: RabGAP AS160/TBC1D4 deficiency increases long-chain fatty acid transport but has little additional effect on obesity and metabolic syndrome in ADMSCs-derived adipocytes of morbidly obese women
Source: Front Mol Biosci. 2023 Aug 3;10:1232159. doi: 10.3389/fmolb.2023.1232159 (PMC10435366; doi:10.3389/fmolb.2023.1232159)
Supplement: Supplementary file 2 [file DataSheet2.docx]

**Supplementary materials**

**RabGAP AS160/TBC1D4 deficiency increases long-chain fatty acid transport but has little additional effect on obesity and metabolic syndrome in ADMSCs-derived adipocytes of morbidly obese women**

**Agnieszka Mikłosz 1*, Bartłomiej Łukaszuk 1, Elżbieta Supruniuk 1, Kamil Grubczak 2, Magdalena Kusaczuk 3, Adrian Chabowski** 1

***Correspondence:** Agnieszka Mikłosz, PhD,

**The primers used for real-time PCR.**

| **Target gene** | **Forward primer (5’-3’)** | **Reverse primer (5’-3’)** |
| --- | --- | --- |
| **TBC1D4** | AGCTCCAGTGAACAGTGCAGTG | CACTTAGGGACTCATTGCTGC |
| **CD36/SR-B2** | GGTACAGATGCAGCCTCATT | AGGCCTTGGATGGAAGAACA |
| **FATP1** | GCTAAGGCCCTGATCTTTGG | CCAAGTCTCCAGAGCAGAAC |
| **FATP4** | TGGCGCTTCATCCGGGTCTT | CGAACGGTAGAGGCAAACAA |
| **RPL13A** | CTATGACCAATAGGAAGAGCAACC | GCAGAGTATATGACCAGGTGGAA |
